# The Impact of the COVID-19 Pandemic on Barrett’s Esophagus and Esophagogastric Cancer

**DOI:** 10.1053/j.gastro.2021.01.208

**Published:** 2021-05

**Authors:** Richard C. Turkington, Anita Lavery, David Donnelly, Victoria Cairnduff, Damian T. McManus, Helen G. Coleman

**Affiliations:** 1 Patrick G Johnston Centre for Cancer Research, Queen’s University Belfast, Belfast, Northern Ireland; 2 The Northern Ireland Cancer Registry, Queen’s University Belfast, Belfast, Northern Ireland; 3 Centre for Public Health, Queen’s University Belfast, Belfast, Northern Ireland; 4 Department of Pathology, Royal Victoria Hospital, Belfast Health and Social Care Trust, Belfast, Northern Ireland

**Keywords:** Barrett's Esophagus, Esophageal Cancer, Gastric Cancer, Diagnostic and Therapeutic, Endoscopy, BE, Barrett’s esophagus, BSG, British Society of Gastroenterology, EG, esophagogastric, NIBR, Northern Ireland Barrett’s Registry, NICR, Northern Ireland Cancer Registry, SNOMED, Systemized Nomenclature of Medicine

The COVID-19 pandemic has dramatically impacted gastroenterology services worldwide. As coronavirus infection rates rose, many professional bodies advised that all endoscopy, except emergency and essential procedures, be stopped immediately.^1^^,^^2^ Upper gastrointestinal endoscopy was considered a high-risk procedure due to a greater potential for aerosolization and transmission of the SARS-CoV-2 virus.^2^ The resulting decline in endoscopic activity has been swift and profound. Markar et al^3^ demonstrated that by April 2020, activity was more than 90% lower than the previous year in 68% of health trusts in England, with an estimated 750 esophagogastric (EG) cancers undiagnosed. In the United States, 98.6% of centers postponed all elective endoscopies for a mean of 5.8 weeks, with remaining uncertainty on how to address the backlog.^4^ The British Society for Gastroenterology (BSG) guidance on restarting endoscopy in the deceleration and early recovery phase of the pandemic continues to advise against surveillance endoscopy, with capacity reserved for urgent procedures.^5^

We aimed to describe the impact of the COVID-19 pandemic on the pathologic diagnosis of Barrett’s esophagus (BE) and EG cancer within population-based databases in Northern Ireland.

## Materials and Methods

The Northern Ireland Cancer Registry (NICR) is a population-based register covering approximately 1.9 million inhabitants and all 4 pathology laboratories in the region. Ethical approval for the NICR, including the waiving of the requirement for individual patient consent, was granted by the Office for Research Ethics Committees of Northern Ireland (ORECNI reference 20/NI/0132).

Electronic pathology reports were received by the NICR and used to identify all unique patients diagnosed with histopathologically confirmed EG cancer (corresponding to International Classification of Disease, 10th Revision codes C15 and C16), or BE (corresponding to Systemized Nomenclature of Medicine (SNOMED) location codes T56010 or T56000 in combination with morphology codes D530910 or M73320), between March 1, 2020, and September 12, 2020 (weeks 10–37). Data were compared with the 3-year average number of histopathologically confirmed patients during the same time period between 2017 and 2019. Further information is available in the Supplementary Materials and Methods.

## Results

**Figure 1:**
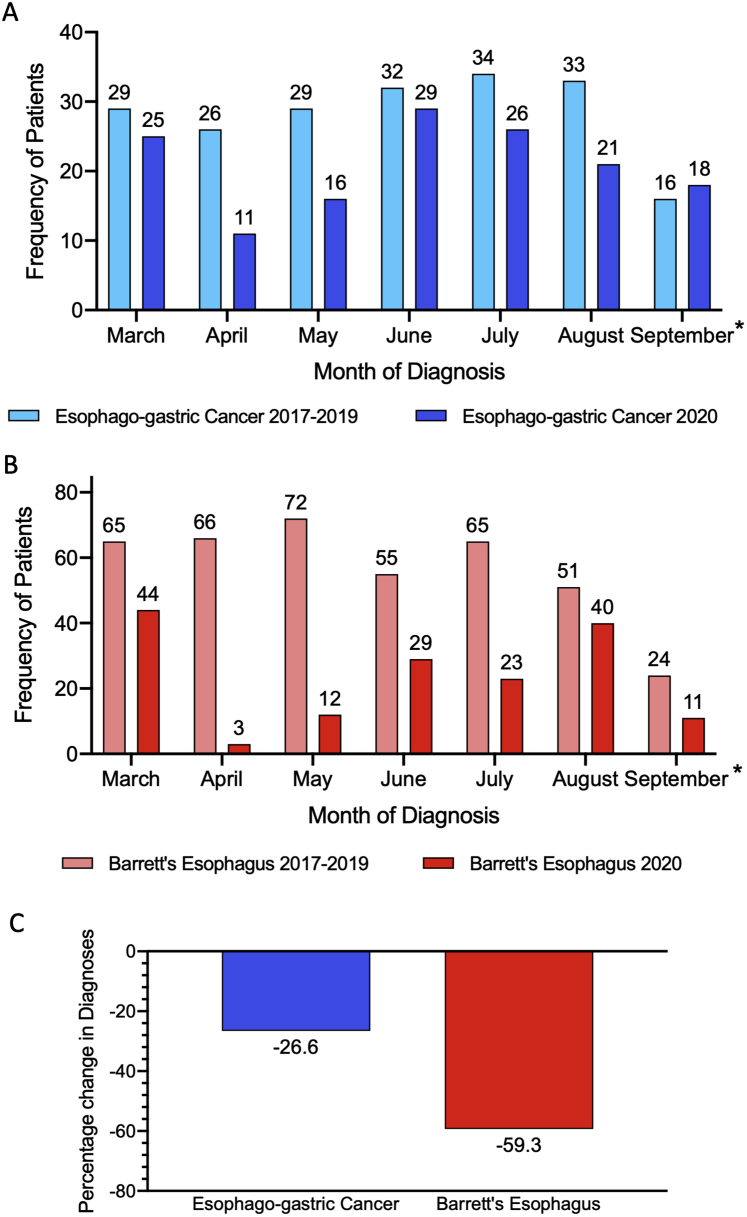
Frequency of (*A*) esophagogastric cancer and (*B*) Barrett’s esophagus diagnoses per month in 2020 compared with the monthly average for 2017 to 2019. (*C*) Percentage decline in esophagogastric cancer and Barrett’s esophagus diagnoses for the period of March to September 2020. ∗Data only available until the week ending September 12, 2020.

Between March and September 2020, the proportion of EG cancer diagnoses declined by 26.6%, compared with the equivalent time frame in 2017 to 2019 (Figure 1*A* and *C*). There was evidence of recovery in the summer months, with diagnoses in the first half of September returning to expected levels. In total, 53 fewer EG cancer cases than expected were diagnosed between March and September 2020.

**Supplementary Figure 1:**
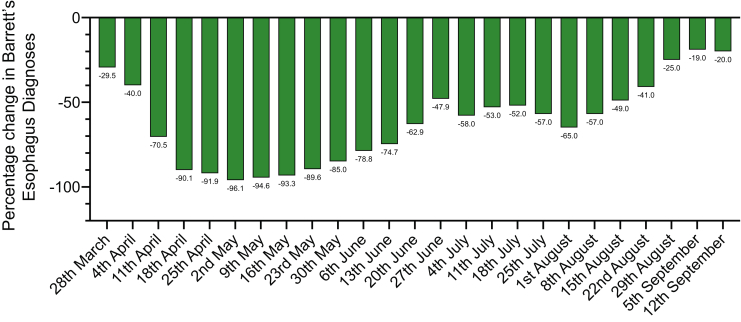
Weekly percentage change between 2017-19 and 2020 in patients whose first pathology sample indicating Barrett's esophagus was taken in the previous five weeks (week end dates represented).

The proportion of BE diagnoses declined by 59.3% compared with the equivalent time frame in 2017 to 2019 (Figure 1*B* and *C*). Notably, in April, only 3 unique patients had a BE diagnosis in Northern Ireland, representing a 95.5% decline in diagnoses compared with previous years, with a maximal weekly decline of 96.1% (Supplementary Figure 1). There was limited evidence of recovery in the summer months, with BE diagnoses remaining 20% below expected levels at the end of the study period. In total, 236 fewer BE cases than expected were diagnosed between March and September 2020.

## Discussion

We have demonstrated that during the first 6 months of the COVID-19 pandemic, pathologic diagnoses of BE fell by 59.3% compared with historical rates, with a 95.5% decline in April alone. The suspension of endoscopy services, disruption to clinical activity, and decline in presentation of symptomatic patients also led to a 26.6% fall in EG cancer diagnoses.

Our study represents the first report to quantify the impact of the COVID-19 pandemic on pathological diagnoses of BE. The BSG advised that all endoscopy, except emergency and essential procedures, be stopped immediately, resulting in the suspension of BE surveillance,^1^ while the guidelines from American gastroenterology professional societies commented that surveillance or treatment of premalignant conditions should not be delayed.^6^

Worldwide, these guidelines have led to variations in practice, but other contributing factors may include local service pressures such as staffing levels and the availability of personal protective equipment. Further updated guidance released by the BSG during the deceleration and early recovery phase of the first wave of the pandemic recommended that surveillance of BE remain suspended.^5^ This is illustrated by our data, which have quantified the slow recovery in BE diagnoses, with rates remaining below their historical baseline.

An important strength of our study is its population-based data from Northern Ireland. However, caution is required over the identification of unique patients and data stability due to reporting delays and the use of pathological BE diagnoses detected by SNOMED codes compared with the more accurate curation methods used by the Northern Ireland Barrett’s Registry (NIBR). The NIBR is a population-based registry of all patients diagnosed with columnar-lined esophagus in Northern Ireland since 1993.^7^ The detailed data extraction undertaken by the NIBR was not feasible for the rapid reporting of BE cases. Comparison of the SNOMED coding used here with NIBR data for 2016 to 2018 indicates that SNOMED coding will detect approximately two-thirds of BE cases (VC 2020, personal communication). Therefore, we are likely to be underestimating the absolute number of cases; however, the proportional decline in BE diagnoses likely remains the same.

Efforts to mitigate the effects of COVID-19 on endoscopy services are ongoing. Recommendations on best practice have been rapidly instituted worldwide to limit SARS-CoV-2 infections in patients and health care workers.^2^^,^^5^ The introduction of nonendoscopic strategies, such as the use of the Cytosponge device (Medtronic, Minneapolis, MN), have also been suggested to triage patients with mild-to-moderate dysphagia.^8^ Implementation of these procedures has been challenging, and the preservation of endoscopic activity during subsequent waves of the pandemic will require ring-fenced resources to prevent further disruption to diagnostic services.

The disruption to BE surveillance may have long-term clinical consequences. The risk of progression of nondysplastic, short-segment BE is low, and so a 6-month or more delay may not be a major risk for this patient group. However, for other higher-risk patients, the effect of the suspension of BE surveillance programs may be more substantial.^7^ Detailed follow-up will be required to assess for changes in dysplasia or cancer incidence in the BE surveillance population in the future.

## Conclusion

We have shown the profound impact of COVID-19 on EG cancer and BE, with a marked fall in pathologic diagnoses in the initial stages of the pandemic. Although the diagnosis of EG cancer shows some signs of recovery, BE detection and monitoring continues to lag behind expected rates. It is imperative that endoscopic services are protected during subsequent waves of the pandemic to preserve the ability to rapidly detect and diagnose cancer and premalignant conditions.

## Supplementary Materials and Methods

### The Northern Ireland Cancer Registry

The NICR has collected information on all patients diagnosed with cancer and certain premalignant conditions in Northern Ireland since 1993. The NICR is a population-based register covering approximately 1.9 million inhabitants, is the officially recognized provider of cancer statistics for Northern Ireland, and has robust validity against key performance indicators of high-quality cancer registration.^1^ Ethical approval for the NICR databases, including the waiving of requirement for individual patient consent, was granted by the Office for Research Ethics Committees of Northern Ireland (ORECNI reference 20/NI/0132).

### Esophagogastric Cancer Diagnoses

Electronic pathology reports were received by the NICR and used to identify all unique patients diagnosed with EG cancer (corresponding to International Classification of Disease, 10th Revision, codes C15 and C16), and histopathologically confirmed between March 1, 2020, and September 19, 2020, in Northern Ireland. These data were compared with the 3-year average number of patients with a pathologic diagnosis of EG cancer during the same time period (corresponding to weeks 10–37) between 2017 and 2019.

### Barrett’s Esophagus Diagnoses

SNOMED codes were used to identify patients with BE diagnoses in Northern Ireland between March 1, 2020, and September 19, 2020, and the same time period for the years 2017 to 2019. Location codes T56010 (esophageal mucous membrane) or T56000 (esophagus) were used in combination with morphology codes D530910 (Barrett’s esophagus or Barrett’s metaplasia) or M73320 (intestinal metaplasia), as advised by an expert gastrointestinal histopathologist (D.Mc.M.).

### Data Analysis

Descriptive statistics (frequencies and proportions over time) are presented for the number of patients diagnosed with BE and EG cancer in Northern Ireland between March and September 2020, respectively. Comparisons were made to the same week range for 2017 to 2019, for which a 3-year average was estimated.
